# Familial multiple lipomatosis associated with multiple cherry hemangiomas and moles: a rare case report

**DOI:** 10.1093/jscr/rjae037

**Published:** 2024-02-06

**Authors:** Gopalaswamy Guntupalli, Rithika Ramadugu, Tarun K Suvvari, Shivani Ravipati, Vimal Thomas

**Affiliations:** Department of Surgery, Kamineni Academy of Medical Sciences and Research Centre, LB Nagar, Hyderabad, Telangana 508254, India; Department of Surgery, Kamineni Academy of Medical Sciences and Research Centre, LB Nagar, Hyderabad, Telangana 508254, India; Rangaraya Medical College, Kakinada, Andhra Pradesh 533001, India; Department of Surgery, Dr. Pinnamaneni Siddhartha Institute of Medical Sciences & Research Foundation, Gannavaram, Andhra Pradesh 521286, India; Tbilisi State Medical University, Tbilisi 0186, Georgia

**Keywords:** familial multiple lipomatosis, genetics, hemangiomas, moles, lipomas, case report

## Abstract

Lipomas are common benign mesenchymal tumours, whereas lipomatoses are uncommon. Familial multiple lipomatosis (FML) is a rare syndrome characterized by multiple usually painless lipomas which may be associated with other conditions. FML is considered to be genetic, with various patterns of inheritance suggested. In this case report, we described a case of multiple familial lipomatosis that was misdiagnosed as dercum’s disease.

## Introduction

Lipomas are common soft tissue tumours of the adipose tissue, classified under lipodystrophies [1]. Presence of many lipomas in a patient accounts to lipomatosis. Familial multiple lipomatosis (FML) is a rare condition that presents with multiple subcutaneous adipose deposits usually in the form of lipomas distributed over the body [2]. It is a relatively rare disease with prevalence of 1/50 000(0.002%) [3]. There have been cases of FML with autosomal dominant inheritance pattern [3, 4]. Adipose tissue tumours can be classified into solitary/sporadic lipomas, FML, and multiple symmetric lipomatosis [5]. FML and multiple symmetric lipomatosis were studied in detail by Lemaitre *et al.* [6]. FML has been associated with various benign and malignant tumours and few gastrointestinal diseases. So, we present a case of FML with cherry angiomas associated with atypical mole syndrome.

## Case report

A 48-year-old woman presented with multiple subcutaneous masses over her body, which were concentrated around her arms, hands, shoulders, back, and thighs. The patient observed the initial mass when she was ~24 years old, the masses reportedly start off as small masses (1 × 1 cm) and gradually increase in size to ~4 × 4 cm. She reported pain associated with the masses, only during the initial stages and when there is an increase in the size. She reported red moles that are present all over her body with distribution similar to that of the lipomas. Patient reported to be diagnosed with Dercum’s disease (DD) 2 years ago and was managed conservatively.

**Figure 1:**
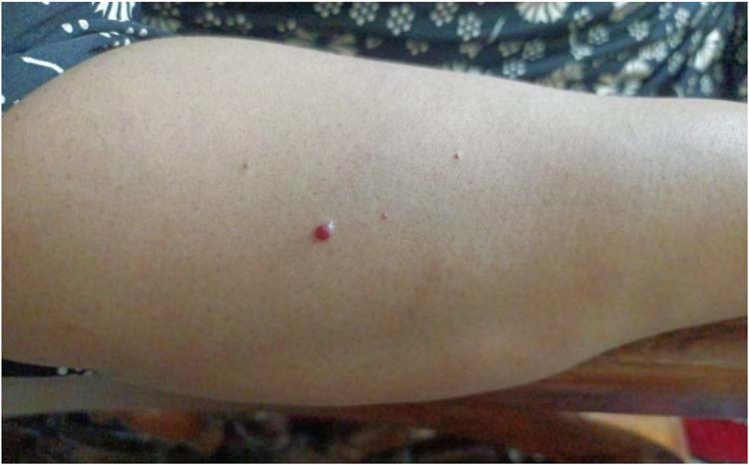
Multiple cherry hemangiomas on the dorsal side of right forearm.

Patient has history of asthma, allergic rhinitis, and dermatitis. Similar complaints were reported in the family. Upon examination, there were ~30 subcutaneous masses on the right upper limb and ~24 on the left upper limb, of various sizes with the largest measuring 4 × 3.5 cm and the smallest measuring 2 × 2 cm. There was no local rise of temperature, tenderness was present over few of the masses, slip sign /slippage sign (gently sliding the fingers off the edge causes the tumours feel to slip out from under—which is most commonly used to differentiate lipomas from cystic swellings) was positive which is suggestive of lipomas. The patient also had multiple cherry hemangiomas (18) and multiple moles (20) with distribution similar to that of the lipomas, that she reported increased in size gradually ranging from 0.05 × 0.05 cm to 0.2 × 0.2 cm (Fig. 1). Over 80 moles were present all over the body, which apparently appeared slowly over her life with one nevus with irregular borders. No cafe au lait spots, freckling or neurofibromas were present. So, a diagnosis of FML was made based on examination findings.

Ultrasound of right forearm revealed multiple lipomas with higher frequency over the arm and lateral border of forearm. Laboratory investigations revealed eosinophilia (4%) and increased FSH (85.90 mIU/mL). Ultrasonography of abdomen revealed grade I fatty liver, multiple large calculi in the gall bladder largest measuring 11 mm, 2 calculi in the right kidney, largest measuring 4 mm. Electrocardiogram (ECG) is normal and 2D echocardiogram showed mild concentric LVH, Grade I diastolic dysfunction, Trivial MR, sclerotic aortic valve with trivial Atrial regurgitation and mild Tricuspid regurgitation.

The patient was managed conservatively upon the patient’s insistence with symptomatic treatment with major focus on pain relief. Further genetic testing: cytogenetics and exome sequencing was suggested to detect the presence of any chromosomal aberrations or mutations that might lead to development of any neoplasia.

## Discussion

Lipomas are the most common adipose tissue tumours. On examination, lipomas are globular, soft and show a positive slip sign. Gross appearance is globular shape, yellow in colour, with capsule and septa might be visible. Microscopically, mature adipocyte lobules, central vacuole and a fibrous capsule are observed, with occasional presence of blood vessels (angiolipomas). Initial imagining technique used is ultrasonography (USG), followed by magnetic resonance imaging (MRI) and computed tomography (CT), if necessary and if suspicion of malignancy arises. Presence of multiple lipomas is considered lipomatosis, which could be sporadic or familial. Association has been reported between chronic alcohol use, HIV patients taking antiretrovirals and lipomatoses [7, 8]. Herbst studied subcutaneous adipose tissue disorders as lipedema, FML, angiolipomatosis, DD, multiple symmetric lipomatosis (MSL) with distinct comparisons [9].

FML is a rare inherited form of hereditary lipomatosis reported to have autosomal dominant inheritance pattern though the exact gene responsible has not been identified. Authors have proposed a polygenic inheritance [10]. Ersek *et al.* proposed that it could be a sex-linked disease, dependant on variables [11]. Cytogenic studies of sporadic lipomas (FNAC) have showed chromosomal translocations including rearrangement of 12q13-15 and 6p21 [12, 13]. These translocations usually involve rearrangements of the HMGA2 gene (12q13-15 locus) which is frequently detected in benign mesenchymal tumours [14]. Retinoblastoma gene (RB Gene) mutations have been associated with lipomas [14]. PTEN gene mutation-Cowden syndrome has been associated with various other benign growths like lipomatosis, hemangiomas, hamartomatous polyps, intestinal polyps, and pigmented macules of the glans penis and some malignant tumours affecting breasts, endometrium, and thyroid [15]. PALB2 mutation has been reported in a case of FML with increased predilection to cancers [16]. FML is often misdiagnosed as MSL, DD, neurofibromatosis, and Legius syndrome. Our case was misdiagnosed as DD or adiposis dolorosa which is defined as painful fatty masses and chronic healing cycle disorder. FML presentation may appear similar to generalized nodular type DD that is characterized by intense pain, clustered lipomas of variable size. Thorough history of the patient and family can help differentiate between both. In FML, pain is reported when the lipoma is forming or sudden pain in one of the lipomas, which may spread to other lipomas (lipoma dolorosa). The pain and size of the lipomas decreases with weight loss. Other differences include absence of insulin resistance, presence of moles, cherry angiomas, neuropathy. Lipoma dolorosa syndrome in families with FML is not the same as DD [17].

There has been an association reported between FML and other benign tumours like Angiomas, brain lipomas and neurofibromas and leiomyoma, gastrointestinal lipomatosis, extra gastrointestinal lipomatosis, neoplastic tumours, polyneuropathies, gastrointestinal comorbidities like Cowden's disease, and celiac disease [6].

Treatment of FML includes primarily screening for other associated conditions—benign and malignant through physical examination and investigations. Genetic testing can be suggested to identify the presence of any chromosomal aberrations, mutations of tumour suppressing genes. Lifestyle changes—diet and exercise can be followed to control the obesity can be suggested. If any lipomas are interfering in the daily life of the patient by pain or restricting movements, can be surgically excised. Statins have shown to reduce the size of the lipomas [18]. Further, collagenase injections and deoxycholic acid can also be used to shrink and destroy lipomas [19]. Liposuction is one of most commonly used procedure for lipomatoses with favourable outcomes, and better cosmetic results in the combination which often involves employing liposuction, excision, and tunnelling techniques, utilizing small remote incisions [20]. Additionally, novel methods, such as the experimental use of laser technology have also been explored for lipomas [21].

## Conclusion

FML is a relatively rare disease that could be misdiagnosed, amongst the other lipomatoses. In all patients presenting with more than one lipomas, or have family history of multiple lipomas, FML must be included on the differential diagnosis as some patients present with a small number of lipomas and some patients might not notice the presence of the lipomas. There is a necessity for further studies on the inheritance, streamlining the diagnostic criteria and treatment modalities.
